# Outcome Measures and Biomarkers for Disease Assessment in Takayasu Arteritis

**DOI:** 10.3390/diagnostics12102565

**Published:** 2022-10-21

**Authors:** Durga Prasanna Misra, Neeraj Jain, Manish Ora, Kritika Singh, Vikas Agarwal, Aman Sharma

**Affiliations:** 1 Department of Clinical Immunology and Rheumatology, Sanjay Gandhi Postgraduate Institute of Medical Sciences (SGPGIMS), Lucknow 226014, India; 2 Department of Radiodiagnosis, Sanjay Gandhi Postgraduate Institute of Medical Sciences (SGPGIMS), Lucknow 226014, India; 3 Department of Nuclear Medicine, Sanjay Gandhi Postgraduate Institute of Medical Sciences (SGPGIMS), Lucknow 226014, India; 4 Clinical Immunology and Rheumatology Services, Department of Internal Medicine, Postgraduate Institute of Medical Education and Research (PGIMER), Chandigarh 160012, India

**Keywords:** Takayasu arteritis, outcome measures, disease activity, damage, biomarkers, computed tomography angiography, magnetic resonance angiography, doppler ultrasound imaging, contrast agent, positron emission tomography-computed tomography

## Abstract

Takayasu arteritis (TAK) is a less common large vessel vasculitis where histopathology of involved arteries is difficult to access except during open surgical procedures. Assessment of disease activity in TAK, therefore, relies on surrogate measures. Clinical disease activity measures such as the National Institutes of Health (NIH) score, the Disease Extent Index in TAK (DEI.TAK) and the Indian TAK Clinical Activity Score (ITAS2010) inconsistently associate with acute phase reactants (APRs). Computerized tomographic angiography (CTA), magnetic resonance angiography (MRA), or color Doppler Ultrasound (CDUS) enables anatomical characterization of stenosis, dilatation, and vessel wall characteristics. Vascular wall uptake of 18-fluorodeoxyglucose or other ligands using positron emission tomography computerized tomography (PET-CT) helps assess metabolic activity, which reflects disease activity well in a subset of TAK with normal APRs. Angiographic scoring systems to quantitate the extent of vascular involvement in TAK have been developed recently. Erythrocyte sedimentation rate and C-reactive protein have a moderate performance in distinguishing active TAK. Numerous novel biomarkers are under evaluation in TAK. Limited literature suggests a better assessment of active disease by combining APRs, PET-CT, and circulating biomarkers. Validated damage indices and patient-reported outcome measures specific to TAK are lacking. Few biomarkers have been evaluated to reflect vascular damage in TAK and constitute important research agenda.

## 1. Introduction

Takayasu Arteritis (TAK) is an uncommon variant of large vessel vasculitis (LVV). Young females are most commonly affected by TAK [1,2]. Granulomatous inflammation characterizes the pathology of TAK. TAK more commonly affects the aorta and its major branches [2,3].

Assessment of disease activity is intrinsic to the management of autoimmune inflammatory diseases. Active disease is generally treated with the initiation or intensification of immunosuppressive therapy. Damage refers to the sequelae of previously active diseases which are not amenable to modification by immunosuppressive therapy. In other autoimmune diseases such as rheumatoid arthritis or systemic lupus erythematosus, the sites of pathology (such as joints, skin, and kidneys) are easily accessible for clinical examination or histopathological evaluation. This enables easier distinction between active disease and damage. The sites of TAK pathology are inaccessible for biopsy, except during open surgical vascular procedures. This is quite unlike Giant Cell Arteritis (GCA), the counterpart LVV of TAK, where the temporal arteries (easily accessible for biopsy and histopathological examination) are commonly affected. Therefore, the distinction of active disease is challenging in TAK. Such an assessment often relies on surrogate biomarkers in peripheral blood, such as inflammatory markers (erythrocyte sedimentation rate (ESR), pentraxins including C-reactive protein (CRP)), and populations of circulating cells. Clinical indices are also used to delineate active disease or damage. Angiographic assessment might help to denote active disease, particularly when carried out serially to identify the progression of disease extent. Positron emission tomography-computerized tomography (PET-CT) using 18-fluorodeoxyglucose (18-FDG) or other ligands or PET-magnetic resonance imaging (PET-MRI) is useful to indicate metabolic activity and other disease processes in vivo in the large arteries. Patient-reported outcomes are increasingly being recognized in different rheumatic diseases including TAK. Recently, attempts have been made to combine distinct modalities to derive a composite score to denote disease activity in TAK. A paper from the Outcome Measures in Rheumatology group delineated specific domains indicating disease activity on clinical assessment and imaging common between TAK and GCA or specific for either disease [4]. In this narrative review, we critically analyze the different outcome measures and biomarkers used for disease assessment in TAK and delineate the agenda for further research in this area.

## 2. Clinical Assessment of Disease Activity and Damage

### 2.1. Disease Activity

Clinical assessment of disease activity in TAK relies on a composite assessment of clinical features, inflammatory markers, and serial imaging. A scoring system proposed by the National Institutes of Health (NIH), United States of America (USA) was the first widely accepted measure of disease activity in TAK [5]. Subsequently, the Disease Extent Index in TAK (DEI.TAK) [6] and the Indian TAK Clinical Activity Score (ITAS2010) [7] were developed by vasculitis researchers from India and the United Kingdom (UK). A set of criteria were used for assessing disease activity in clinical trials of GCA and TAK [8].

#### 2.1.1. NIH Score

A seminal paper in 1994 from the NIH proposed active TAK to be determined if at least two of the following four criteria were deemed to be new or worse when compared with the last visit: i. the presence of constitutional features such as fever and arthralgias; ii. ESR more than 20 mm/h; iii. features to suggest vascular involvement, viz., pulse loss, vascular bruits, limb claudication, blood pressure asymmetry between either the upper or the lower limbs, tenderness on palpation of arteries such as carotidodynia; or iv. angiography consistent with vascular involvement of TAK. Smoldering disease was defined as a partial resolution of symptoms or signs suggestive of active TAK. Disease remission was defined as similar or identical symptomatology without demonstrable vascular progression [5].

While the NIH criteria (also eponymously referred to as the Kerr score) for the active disease were logical, these were arbitrary (rather than data-driven) and lacked validation. The values of ESR might vary with gender and age, and can be affected by any disease state that affects fibrinogen levels. Therefore, in the appropriate context, an ESR of >20 mm/h might well be in the normal range [9]. Additionally, the NIH criteria by their definition cannot be used for baseline assessment as they require comparison with a previous visit.

#### 2.1.2. DEI.TAK

The DEI.TAK assesses features in eleven domains that are new or worse in the past 6 months. These domains include systemic features, cutaneous symptoms, mucous membranes, eyes, otorhinolaryngological system, chest, cardiovascular system, abdomen, renal system, nervous system, and genitourinary system. Some cardiovascular features are weighted (bruits, pulse inequality, pulse loss, claudication) as scoring them can lead to a further item to be scored to clearly define the particular feature. The DEI.TAK form also records physician global assessment (PGA) as an active, persistent, or inactive disease. While the scoring for DEI.TAK does not account for ESR or CRP, the values of these are noted in the form [6,10]. The DEI.TAK was derived from the Birmingham Vasculitis Activity Score (BVAS), which is primarily used for small and medium vessel vasculitis [11]. A lot of features on the DEI.TAK, such as cutaneous features (gangrene or cutaneous vasculitis), otorhinolaryngological symptoms, chest symptoms such as respiratory failure, and genitourinary symptoms, are more frequently seen in antineutrophil cytoplasmic antibody (ANCA)-associated vasculitis (AAV). These features are rarely if ever present in TAK [12]. The BVAS separately indicates items that are persistent as opposed to new or worse symptoms. The BVAS also has a ceiling score for each domain. However, the DEI.TAK has no such distinction or ceiling score. The DEI.TAK does not include insights from angiographic assessment into the scoring [6].

The DEI.TAK was validated in a Turkish cohort of 144 patients with TAK. At the initial assessment, all the patients had a DEI.TAK of more than 1. This was discordant with PGA, according to which 62% were active, 16.2% had persistent disease and 21.8% had inactive disease. On the last follow-up visit at a mean interval of 27 months, 100 out of 144 patients had a DEI.TAK score of zero. Again, discordance with PGA was observed. Of these 100 patients, 14% had active and 17% had persistent disease as per PGA. For the 44 patients with a DEI.TAK ≥ 1, eight had the inactive disease by PGA. For 119 visits where NIH criteria could be used due to the availability of imaging, the agreement between NIH criteria and DEI.TAK to assess active disease was 94%. However, a poorer agreement was observed between PGA and either the DEI.TAK (68%) or the NIH score (74%) [6]. To date, cut-offs delineating active and inactive TAK based on the DEI.TAK have not been validated.

#### 2.1.3. ITAS2010

The ITAS2010 assesses features in six domains (systemic, abdomen, renal system, nervous system and genitourinary system, and cardiovascular system) that are new or worse in the past 3 months. The scoring of the ITAS2010 is biased towards the cardiovascular domain (which constitutes 33 out of 44 items). Certain items on the ITAS2010 are weighted, five in the cardiovascular domain (bruits, pulse inequality, pulse loss, claudication, carotidodynia) and one each in the renal (diastolic hypertension) and nervous system (stroke) domains. The ITAS2010 mandates scoring all features noted at the first visit as indicative of active disease. The ITAS2010 form, similarly to the DEI.TAK, also records PGA as an active, persistent, or inactive disease. The scoring for ITAS2010 modified for acute phase reactants (ITAS-A) accounts for ESR or CRP to add values ranging from 0 to 3 to the ITAS2010 score. While the ITAS2010 form notes information from recent angiographic imaging, this is not included anywhere in the scoring [7]. Similar to the DEI.TAK, the ITAS2010 neither distinguishes persistent symptoms from new or worse symptoms nor has a ceiling score for each domain [6,7,10].

The ITAS2010 was validated in a cohort of 132 TAK patients from India. The correlation of ITAS2010 with PGA was moderate for the first visit (r = 0.51). The correlation with PGA increased with subsequent visits (second visit, r = 0.64; third visit, r = 0.74). The correlation of ITAS2010 with the BVAS was strong for the first visit (r = 0.75) and less so for subsequent visits (second visit, r = 0.55; third visit, r = 0.69). ITAS2010 scores only had a weak correlation with ESR (r = 0.22) or CRP (r = 0.18, not statistically significant). Unlike the DEI.TAK, concordance with PGA had not been reported for ITAS2010. Cut-offs of ITAS2010 > 1 and ITAS-A > 4 to denote active disease were proposed arbitrarily. These cut-offs might be problematic. Features such as hypertension can easily be misidentified as active disease whereas they might denote poor compliance with anti-hypertensive agents or simply be a consequence of missing the morning dose of anti-hypertensive on the day of the clinic visit. Arbitrarily scoring all features present at the first visit as active disease might artificially inflate the ITAS2010 at the initial visit. Such features would inevitably not be scored at subsequent visits, therefore appearing as if the disease activity has dramatically reduced. This might explain the poorer correlation of ITAS2010 with PGA at the initial visits [7].

A study from Turkey evaluated the ITAS2010 and ITAS-A in 144 patients with TAK over 289 visits. In this study, the agreement between ITAS2010 and PGA was 66.4%, similar to ITAS-A (67%). The agreement between ITAS2010 and NIH scores was 82.8%, slightly improving when ITAS-A was used instead (86.3%). On serial angiographic assessment, 15 patients demonstrated angiographic progression. All these fifteen had active disease as per the PGA; however, only one of them was deemed active by the ITAS2010. Thus, the ITAS2010 had a poor predictive value for the angiographic progression of TAK [13]. Another study evaluated the combination of angiographic findings with ITAS2010 or ITAS-A in an attempt to improve its ability to detect active TAK. The authors arbitrarily devised a Rad-Active score, which would be considered to denote active disease if any of the following three features were present: involvement of new vessels by any imaging modality, arterial wall thickening demonstrable on ultrasound, or arterial wall contrast uptake or edema demonstrable on magnetic resonance angiography (MRA). In a cohort of 52 TAK patients over 410 visits, Rad-Active had better agreement with PGA (76%) than ITAS2010 (69%). Rad-Active also had better agreement with NIH scores (83%) than ITAS2010 (78%). Furthermore, the authors proposed the ITAS-A-Rad score by adding to the ITAS-A five points for the involvement of new vessels by any imaging modality or three points each for arterial wall thickening demonstrable on ultrasound or arterial wall contrast uptake or edema demonstrable on MRA. The ITAS-A-Rad score had better agreement with PGA (72%) and NIH scores (82%) than the ITAS2010 alone [14].

#### 2.1.4. Other Criteria for the Assessment of TAK Disease Activity

Randomized controlled trials (RCTs) are few and far between in TAK. The trial of abatacept in TAK was the first high-quality RCT in TAK [8,15]. In this trial, patients with TAK were considered to be active if they had fever or symptoms suggestive of vascular involvement or vascular ischemia or had any other symptoms consistent with TAK. Myalgia, arthralgia, fatigue, or malaise if present together with an elevated ESR (>40 mm/hour) or CRP above the reference range were considered to denote active disease. Isolated ESR or CRP elevation without clinical symptoms was not considered to denote disease activity. New onset vascular stenosis or dilatation on angiography also denoted active disease. Some studies have referred to these criteria as the Abatacept in Giant Cell Arteritis and Takayasu arteritis (AGATA) criteria [8]. These criteria appear to have been derived from the NIH scores for TAK disease activity [5]. Modifications to account for higher cut-offs for ESR, the inclusion of CRP, and to denote the implications of isolated ESR or CRP elevation without clinical features of TAK disease activity likely improve the specificity of the NIH criteria. However, this finding has not been objectively validated yet.

Another set of criteria to assess TAK disease activity was proposed by Mexican researchers (eponymously referred to as the Dabague criteria). These criteria score different manifestations of TAK as 0.5, 1, 2, or 3. A score of ≥5 denotes active TAK. These disease activity criteria have not been used much in clinical studies [16].

The European Alliance of Associations for Rheumatology (EULAR) has recently proposed criteria to denote active LVV. These include the presence of signs and symptoms of active TAK or GCA (in the relevant context) along with one of these three features: disease activity evident on vascular imaging or histopathology, vascular ischemia due to disease, or persistent elevation of ESR and CRP without any other explainable cause. The same paper also proposed the use of relapses rather than flares. Major relapses were defined as either distal vascular ischemia or imaging evidence of the new onset of arterial stenosis, dilatation, or dissection. Minor relapses were those relapses not meeting the criteria for major relapses. Remission was defined as quiescent clinical features of the disease, normal inflammatory markers along with angiographic evidence of disease stabilization. If remission was present for at least 6 months without requiring an excess of corticosteroids, it could be called sustained remission. Sustained remission without the requirement for corticosteroids was labeled as glucocorticoid-free remission. Disease unresponsive to standard therapy was defined as refractory disease [17].

It is reasonable to use future relapses as a surrogate measure of disease activity [15]. Both the notable RCTs in TAK (of abatacept and tocilizumab) used the duration of relapse-free survival as their primary outcome [8,18]. Similarly, the percentage of reduction in corticosteroid dose from baseline could also be used as an outcome measure indicative of the attainment of remission or steroid-sparing efficacy of immunosuppressive agents [15].

### 2.2. Damage

Damage refers to a clinically assessed item related to the sequelae of a previously active disease that is not amenable to recovery. When an item is labeled as damaged in the context of immune-mediated inflammatory diseases including vasculitis, it generally indicates that intensification of immunosuppressive treatment is not indicated for that particular disease manifestation. The Vasculitis Damage Index (VDI) was developed in the context of small and medium vessel vasculitis [19]. The TAK Damage Score (TADS) is another tool used to quantify vascular damage in TAK.

#### 2.2.1. Vasculitis Damage Index

The VDI was designed by vasculitis researchers from the UK to capture damage items in different organ systems affected by vasculitis, including drug-related damage [19]. These features should have been present for at least three months to be scored. Very few of the actual items captured on the VDI are directly applicable to TAK. These include i. under the Cardiovascular system: angina/angioplasty, first or recurrent myocardial infarction, cardiomyopathy, hypertension; ii. under the Peripheral vascular disease: pulse loss, vascular stenosis, claudication; iii. under the Gastrointestinal system: mesenteric ischemia and gut infarction; iv. under the Renal system: GFR < 50% of the expected or end-stage renal disease; v. under the Neurological system: stroke; and vi. under others: Diabetes, malignancy or other. Thus, out of 64 items, only 17 might have relevance to TAK [19]. Few studies have used the VDI to document damage in TAK [20,21]. A modified version of the VDI is used to assess damage in vasculitis occurring in childhood [22]. A more extensive damage index, the Combined Damage Assessment Index (CDA), was also developed by updating the VDI items based on information gathered from clinical trials of AAV [23]. However, the CDA is scarcely used in patients with TAK [24]. Akin to the VDI, most items on the CDA are not directly applicable to TAK or LVV.

#### 2.2.2. Takayasu Arteritis Damage Score

The TADS was developed by vasculitis researchers from India and the UK who also developed the DEI.TAK and ITAS2010. Some of the features of the TADS were derived from the VDI. The TADS has not been formally published and validated. The first paper to describe the TADS also provided the scoresheet and glossary for the same [10]. The TADS records features in different domains which have been present for at least six months. The TADS accounts for vascular procedures, including restenosis and the requirement for repeated vascular procedures [10]. Vascular procedures logically indicate vascular damage. However, restenosis and the requirement for repeated vascular procedures for the same vascular territory could very well indicate active disease.

Analysis of domains and specific manifestations scored in the TADS reveals that a lot of features are common between the DEI.TAK and ITAS2010 with TADS (Table 1) [7,10]. Thus, the TADS risks being iterative and might artefactually denote items scored as activity initially and damage later on during follow-up visits. While the VDI categorically states that items should not be scored as damage on the first visit [19], the glossary for the TADS does not clarify this point [10]. Additionally, certain features recorded on the TADS are rarely seen clinically in TAK. Cord lesions are seen in systemic lupus erythematosus or rarely in AAV, but hardly ever in TAK [25,26]. Similarly, respiratory failure may be seen in AAV but is very unlikely to be ever encountered due to TAK, except during pneumonia which is unrelated to TAK [27]. Items such as pulse loss or vascular bruits need not always indicate vascular damage. These features could be due to inflammatory arterial wall edema (indicating active TAK) or vessel wall fibrosis as a consequence of prior vessel wall inflammation (indicating damage). Pulse loss reverses in between a third and one-half of patients following immunosuppressive therapy [28]. Therefore, before labeling pulse loss or bruits as damage, the authors feel that it might be necessary to observe these features on successive visits (at least three consecutive visits). There remains an unmet need for a data-driven clinic damage score for TAK.

The various scoring systems to assess disease activity and damage in TAK are summarized in Table 2.

### 2.3. Patient-Reported Outcome Measures

The importance of patient-reported outcome measures (PROMs) is increasingly being recognized in different chronic diseases. A recent systematic review critically assessed the prevalent literature related to PROMs in TAK. Most of the studies had assessed the quality of life, and few studies had assessed disability, mood disturbances, fatigue, perceptions of illness, and fibromyalgia in TAK. Studies had only reported longitudinal changes in quality of life and disability but not in other PROMs in TAK. Fewer studies had compared PROMs between active and inactive TAK, or assessed changes in PROMs following the initiation of medical treatment. Despite TAK being way more frequent in Asia, very few studies from Asia had assessed PROMs in TAK [29].

There does not yet exist a PROM specific to TAK [29]. Only one study has attempted to develop a TAK-specific PROM by undertaking qualitative interviews of 31 patients with TAK from the USA and Turkey. The study reported that patients with TAK considered pain or discomfort to be of importance during both active and inactive diseases. Fatigue or low energy levels appeared to be important considerations during active disease only. The emotional impact of the disease was important during periods of inactive disease [30]. A TAK-specific PROM that is developed in the future should also consider including these features as well as clinical features of TAK such as claudication and limitation of work capacity due to heart failure [29]. Clinical features related to TAK that are considered important by patients might differ in different geographical regions. Therefore, these might require reassessment by qualitative research in different patient groups [29].

## 3. Angiographic and Imaging Outcomes in TAK

Vascular imaging in TAK utilizes computerized tomographic angiography (CTA), MRA, conventional angiography or digital subtraction angiography (DSA), color Doppler Ultrasound (CDUS), PET-CT, or PET-MRI. While CDUS (including contrast-enhanced ultrasound) has considerable inter-observer variability, CTA, MRA, DSA, and PET-CT/PET-MRI are more objective measures. Qualitatively, the assessment of either improvement or stabilization of vascular stenoses or dilatation on serial follow-up can indicate whether TAK is active or inactive [15,31]. The diagnostic performance of these different modalities for TAK varies. From a recent systematic review, CDUS had a sensitivity of 81% (95% CI 69–89%, three studies) and a specificity of 86% (95% CI 75–92, five studies), MRA had a sensitivity of 92% (95% CI 88–95%, five studies) and a specificity of 92% (95% CI 85–96%, five studies), and PET-CT had a sensitivity of 81% (95% CI 69–89%, 10 studies) and a specificity of 74% (95% CI 55–86%) for diagnosing TAK. Based on a single study, CTA had a sensitivity of 95% and a specificity of 100% for diagnosing TAK [32]. A few scoring systems were devised to denote the extent or activity of vascular involvement in TAK.

### 3.1. CT Angiography

CTA using intravenous contrast is commonly used to evaluate the extent of vascular involvement in TAK through the identification of stenosis, dilatation, and collateral channels for circulation. CTA might also reflect vascular disease activity. On serial follow-up imaging, a decrease in clinically assessed disease activity in TAK was associated with an increase in mural attenuation and vascular wall calcification (in the pre-contrast phase), a reduction in mural thickening and mural enhancement in the contrast-enhanced phase, and fading of the venous phase low-attenuation ring [33,34]. Another study of 19 patients with TAK where the aortic calcium score was quantified reported greater aortic calcification on CT with older age or longer duration of disease. Higher aortic calcium scores were also associated with atherosclerosis [35].

Pericoronary adipose tissue (PCAT) and periaortic adipose tissue (PAAT) are identifiable on coronary CTA. The relationship of these parameters with TAK disease activity has been recently evaluated. Median PCAT was moderately correlated with ESR, CRP, and ITAS-A. Active and inactive TAK could be well distinguished by median PCAT (AUC 0.82, 95% CI 0.70–0.92) but not by PAAT (AUC 0.63, 95% CI 0.45–0.79) [36].

### 3.2. MR Angiography

MRA, usually with intravenous contrast administration, is a useful modality to evaluate arterial wall characteristics which can reflect disease activity in TAK and GCA, apart from the anatomical correlates of stenosis, dilatation, and collateral channels. The anatomical information from MRA can be obtained even without the administration of intravenous contrast, which is useful in patients with renal failure where contrast cannot be administered [1,37]. Wall thickening, wall edema, and mural enhancement are features that suggest active LVV [38]. A reduction in each of these parameters was demonstrated on serial MRA before and after the administration of biologic DMARDs in a cohort of eight patients with TAK and four with large vessel GCA [38]. Three-dimensional contrast-enhanced MRA (CEMRA) has excellent diagnostic accuracy for the detection of any stenotic vascular lesion (92.97%) or vascular stenosis over 50% (97.52%) when compared with the gold standard of DSA [39]. A qualitative assessment of active vasculitis in 20 patients with TAK with CEMRA when evaluated against clinically assessed active disease revealed moderate agreement of 80% (kappa coefficient 0.52) [40].

A scoring system was proposed to quantitate vessel wall imaging changes on delayed CEMRA in TAK. This involved the assessment of stenosis, vascular wall thickening, or vascular wall enhancement each on a grade of 1–3. In the first study assessing this CEMRA scoring system in 26 patients with TAK (sixteen with active disease) across 14 arterial segments, each of these parameters was significantly higher in active than in inactive TAK. Each of the three parameters individually demonstrated a moderate correlation with CRP, platelet counts, and plasma fibrinogen levels [41]. Another study of 52 patients with TAK (30 with active disease) used this same CEMRA scoring system across 12 arterial segments. All three parameters were individually higher in active than in inactive TAK. Each of these parameters moderately correlated with NIH scores and ITAS2010. Stenosis and vascular wall thickening (but not vascular wall enhancement) individually had a weak to moderate correlation with ESR, CRP, pentraxin-3, and platelet counts. On serial imaging in 15 patients with active TAK at an interval of 6 months, the scores for stenosis and vascular wall thickening remained similar, whereas vascular wall enhancement scores came down [42].

Another study compared the diagnostic performance of various parameters on delayed CEMRA (wall thickening, wall edema, early enhanced signal intensity ratio, and pulsed wave velocity) with ESR and CRP for detecting disease activity in 52 patients with TAK (23 with active disease). Each of these parameters (wall thickening AUC 0.804, 95% CI 0.667–0.941; wall edema AUC 0.753, 95% CI 0.587–0.919; early enhanced signal intensity ratio AUC 0.723, 95% CI 0.565–0.880; pulsed wave velocity AUC 0.723, 95% CI 0.566–0.879) fared worse than either ESR (AUC 0.851, 95% CI 0.733–0.969) or CRP (AUC 0.808, 95% CI 0.647–0.969) to distinguish active from inactive TAK [43]. A further study of 27 TAK patients (15 with active disease) reported that delayed CEMRA using a fast low-angle shot sequence was able to distinguish active from inactive TAK [44].

A recent study evaluated the performance of diffusion-weighted imaging MRA without contrast against CEMRA to identify active TAK. In a cross-sectional study of 40 patients with active TAK, low b-value diffusion-weighted imaging had a similar ability to detect active TAK as delayed enhancement T1-weighted imaging on CEMRA [45].

### 3.3. Angiographic Scoring Systems

An angiographic scoring system for TAK and GCA based on either CTA or MRA was proposed by researchers from the UK and Europe. This scoring system provided the Angiographic Composite Score (ACS) derived by summating the Angiographic Stenosis Score (ASS) and Angiographic Dilatation Score (ADS). Both the ASS and ADS were scored across seventeen arterial segments and then summated. The scoring accounted for the percentage of stenosis (for ASS—scores ranging from one to four) or dilatation (for ADS—scores ranging from one to three), and the length of vascular involvement (for both ASS and ADS, scores ranging from one to four). The reliability of the scores was excellent (intra-class correlation coefficient >0.99). ACS and ASS were higher for TAK than for large-vessel GCA. Conversely, ADS was higher for large vessel GCA. At the baseline assessment, ACS and ASS correlated strongly with TADS but not with ESR, CRP, or NIH scores in TAK. Changes in the ASS, ADS, and ACS correlated with increases in disease activity scores (NIH, ITAS2010, and ITAS-A). Changes in the scores predicted the angiographic progression well. One unit change in ASS (AUC 0.949, 95% CI 0.883–0.995), ADS (AUC 0.988, 95% CI 0.972–0.998) and ACS (AUC 0.996, 95% CI 0.990–0.999) reflected the progression of stenosis, dilatation, or overall angiographic progression well [46].

The Combined Arteritis Damage Score (CARDS) was derived from 41 TAK and 55 GCA patients from the UK based on information from CTA/MRA and PET-CT. Across 25 arterial segments, mild stenosis, moderate to severe stenosis, vascular occlusion, and vascular dilatation were given numerical attributes and summated. CARDS was higher for TAK than for GCA. The presence of constitutional symptoms or the use of biologic DMARDs was associated with lower CARDS, whereas a longer disease duration was associated with higher CARDS on multivariable-adjusted analyses [24].

### 3.4. Color Doppler Ultrasound

One of the earliest systematic descriptions of CDUS in TAK reported uniform wall thickening of involved arteries to be characteristic of TAK [47]. Since then, ultrasound was reported as one of the imaging modalities for disease assessment in TAK while becoming the imaging modality of choice for the temporal arteries in GCA. A gradual transition of intima-medial thickness from areas of normal to abnormal arterial wall (the “slope” sign) might enable the distinction of arteritis from atherosclerosis (where the transition is much more abrupt). However, vasculitis might co-exist with atherosclerosis in the same vessel. This sign was described for GCA but has yet to be validated in TAK [48,49,50].

The CDUS TAK score from Kolkata (CDUS-K) attempted to quantify the extent of arterial involvement in TAK using CDUS to assess nineteen arterial segments. Each segment was scored as 0 or 1 depending on the flow pattern on CDUS—0 if there was normal triphasic flow and 1 if there was biphasic, monophasic, or no flow (each of which indicates vascular stenosis). The CDUS-K was assessed in 19 TAK patients and compared against angiographically demonstrable stenosis and ITAS2010 scores. Good agreement was observed between the CDUS-K and angiography (kappa coefficient ranging from 0.638 to 0.905 for different vascular territories, overall 0.725, 95% CI 0.488–0.961). There was a moderate correlation between CDUS-K and ITAS2010 (r = 0.714). A limitation of this study was the lack of assessment of correlation or concordance between CDUS-K and ITAS2010 on follow-up visits [51]. Another scoring system used in GCA is the halo sign, where halos evident on CDUS are scored for the three segments of the temporal arteries and axillary arteries on both sides. Higher scores predict a greater risk of ocular ischemia [52]. Such a halo score valid for TAK may be an area for future research.

Arterial wall inflammation is characterized by neovascularization of the arterial wall from the vasa vasorum [53]. Contrast-enhanced ultrasound (CEUS) relies on the uptake of microbubble contrast in the arterial wall consequent to such neovascularization. CEUS to denote disease activity in the carotid arteries in TAK was first reported nearly a decade ago [54]. Subsequent reports revealed that higher degrees of wall uptake (grade 1—mild to moderate uptake; grade 2—prominent uptake) were associated with active TAK, while lesser degrees of CEUS uptake (grade 0—no or minimal uptake) could also be seen in inactive TAK [55]. A study involving 17 patients of TAK for whom CEUS was performed over 40 assessments (13 during periods of active disease) compared CEUS with carotid intima-media thickness (CIMT). Upon combining CIMT with higher grades of CEUS uptake, active TAK could be better distinguished from inactive TAK (AUC 0.99) when compared to CIMT > 2.7 mm (AUC 0.83) [56]. Another study involving 14 TAK and 17 GCA patients reported an association between the grade of CEUS uptake and PET-CT uptake. CEUS had a sensitivity of 92% and specificity of 100% identifying active arteritis when PET-CT uptake of at least grade 2 assessed visually was considered the gold standard [57]. From another study of 71 patients with TAK, the grade of CEUS uptake had a weak correlation with ITAS2010, ESR, and CRP, and a moderate correlation with NIH scores or PET-CT uptake in the carotids. CEUS had excellent performance (AUC 0.968) to identify active arteritis when PET-CT uptake of at least grade 2 assessed visually was considered the gold standard [58]. In another cohort of 28 patients with TAK, CEUS had an AUC of 0.872 (95% CI 0.785–0.959) to distinguish active TAK [45].

Few studies have assessed the combination of CEUS with other modalities to distinguish active TAK. In a study of 84 patients with TAK (47 of whom were active), CIMT > 1.75 mm or CEUS uptake ≥ grade 2 when combined with ESR > 20 mm/hr had a good performance to detect active TAK (AUC 0.848). On serial CEUS in those with active TAK at baseline, the authors reported a decrease in wall thickness and CEUS grade concomitant with a reduction in disease activity [59]. Some studies have reported contrary results. A study evaluated 86 TAK patients over 159 visits with CEUS (92 visits were during active TAK). They quantified the uptake in the carotid artery following CEUS, which was increased in those with active TAK when compared with inactive TAK. The intensity of CEUS uptake had a good performance to distinguish active TAK (AUC 0.863, 95% CI 0.797–0.929); however, there were no incremental benefits evident on adding ESR, CRP, or CIMT to CEUS [60]. While CEUS appears to be a promising modality to assess TAK disease activity, it can only be assessed in vessels well accessible to ultrasound examination such as the carotid arteries. Its utility for the assessment of disease activity in TAK not involving the carotid arteries is uncertain.

### 3.5. PET-CT and PET-MRI

PET-CT commonly uses 18-FDG to denote areas of metabolic activity in the blood vessels and other body organs. CT is used for attenuation correction and anatomic localization of the metabolic activity on PET. The quantitative evaluation of metabolic activity is carried out by comparing the uptake in a particular region to the background uptake in the liver or mediastinal blood pool. Assessments of arterial wall metabolic activity can be performed visually (active or inactive). Grading could be performed qualitatively in comparison to the liver (0–1 if uptake less, 2 if uptake equal to, 3 if uptake greater than the liver). Semiquantitative techniques include the maximum or mean value of standardized uptake values (SUV_max_ or SUV_mean_) and target-to-blood pool ratio (TBR) [61]. A recent scoring system, the PET vascular activity score (PETVAS), summates the grades of uptake in different arterial segments [62].

One of the earliest descriptions of PET-CT in TAK reported its use in 39 patients with TAK (27 with active disease). Using a SUV_max_ cut-off of 2.1, the AUC to distinguish active TAK was 0.954, better than ESR (0.727) or CRP (0.847) [63]. Another report retrospectively analyzed 60 PET-CTs undertaken in 51 patients with TAK. All the seventeen scans undertaken at baseline assessment suggested active disease. Only 14 of the 43 scans on follow-up after the start of immunosuppressive therapy indicated active disease, demonstrating that PET-CT activity decreases after treatment in TAK [64]. A study of 14 TAK patients on immunosuppressive therapy with persistent elevation of acute phase reactants (APR) despite clinically inactive disease reported at least grade 2 uptake on PET-CT in 9/14 patients [65]. The value of PET-CT on follow-up imaging of TAK remains uncertain. From a study involving 20 TAK patients on immunosuppressive therapy (13 with active disease), PET-CT was judged to be active in 12/13 with clinically active disease and all seven with inactive disease [66].

A seminal paper from the NIH, USA described the first scoring system for PET-CT in LVV based on 56 patients with LVV (26 TAK and 30 GCA, 111 PET scans) compared with 59 control subjects (seven healthy, 17 LVV mimics, and 35 with dyslipidemia, 59 PET scans). A positive PET-CT suggesting active vasculitis was observed in 34/40 LVV with clinically active disease, 41/71 with clinically inactive disease, and 10/59 control subjects. After multivariable adjustment, active disease by clinical assessment, earlier disease duration, lesser body mass index, and lower daily prednisolone dose were associated with greater odds of 18-FDG uptake. However, APRs or immunosuppressive therapy other than corticosteroids did not associate with 18-FDG uptake. Overall, PET-CT had a specificity of 42% (95% CI 31–55%) to distinguish active from inactive vasculitis. Further, 18-FDG uptake in four aortic segments and eleven arterial segments was graded from 0–3. Scores from each of the 15 segments were summated to give the PETVAS. The PETVAS was increased in those with clinically active vasculitis, with moderate performance (AUC 0.72) at a cut-off of ≥20 to distinguish active LVV from remission. In the subset of scans during active LVV, the PETVAS moderately correlated with ESR, CRP, and fibrinogen levels and negatively correlated with prednisolone dose. In those scans conducted during clinical remission, no association with APR or prednisolone dose was observed. In 39 patients who underwent PET-CT during periods of remission, a PETVAS ≥20 predicted a five-fold greater risk of relapse [62].

A subsequent study from the same group assessed serial changes in PET-CT and disease activity (assessed by PGA on a scale of 0 to 10), ESR, and CRP with treatment in 52 LVV (21 TAK and 31 GCA). PETVAS decreased by 6 months with an intensification in immunosuppressive therapy, along with a decrease in PGA, ESR, and CRP. However, qualitative assessment of PET-CT still suggested active vasculitis in 67% on follow-up (vs. 83% at baseline). When there was no change in immunosuppressive treatment over 32 visit intervals, similar values of PETVAS, PGA, ESR, and CRP were observed at both visits. Whenever immunosuppressive treatment was reduced, there was a significant increase in PETVAS without important changes observed in the PGA, ESR, and CRP [67]. Another study compared PETVAS in 50 patients with GCA and 76 with TAK. The PETVAS was higher in GCA than in TAK at baseline assessment. Over follow-up, the PETVAS significantly reduced with time in GCA but not in TAK [68]. A further study from this group compared various quantitative and qualitative measures of LVV activity on PET-CT in 95 patients (43 TAK, 52 GCA) over 206 visits (75 visits during active disease). The authors calculated TBR compared to the liver (TBR_Liver_) or to the blood flow (TBR_Blood_), SUV_Artery_ (by averaging SUV_max_ across nine arterial territories), and the PETVAS at each of these visits. Each of these parameters had moderate performance, comparable with each other, to distinguish active TAK (AUC for TBR_Liver_ 0.66 (95% CI 0.58–0.73) at a cut-off of 1.46, TBR_Blood_ 0.65 (95% CI 0.57–0.73) at a cut-off of 2.39, SUV_Artery_ 0.59 (95% CI 0.51–0.68) at a cut-off of 3.58, and PETVAS 0.65 (95% CI 0.57–0.73) at a cut-off of 22.5) [69]. Another study assessed 100 patients with LVV (49 TAK, 51 GCA) who had experienced 476 PET-CTs over more than 8 years of follow-up. When compared with a clinical assessment of active or inactive disease, qualitative assessment (AUC 0.70, 95% CI 0.65–0.75) had similar performance when compared with PETVAS ≥ 10 (AUC 0.73, 95% CI 0.68–0.79) [70].

Another seminal paper assessed 18-FDG uptake on PET-CT in 26 TAK patients more than 6 months after surgical vascular grafts. Twenty-three of the twenty-six patients had 18-FDG uptake at the graft site (median grade 3). However, serial MRA revealed no progression of arterial involvement in 25 of these 26 patients. Nine of these patients had a subsequent intensification of immunosuppressive therapy. However, on serial imaging, 18-FDG uptake continued to increase at graft sites. This suggested that PET-CT demonstrated artefactual periprosthetic graft uptake [71]. A further paper from the same group assessed 30 patients with TAK from Italy (18/30 active as per the NIH criteria), of whom 16/30 had 18-FDG vascular uptake on PET-CT. ESR and CRP were similar in patients with or without PET-CT uptake. Neither ESR nor CRP correlated with the number of arterial segments with 18-FDG uptake or with the SUV_max_. Thus, 18-FDG uptake on PET-CT seems to identify a proportion of active TAK where the ESR and CRP are normal. Uptake on PET-CT had poor concordance with NIH disease activity scores [72]. From a recent systematic review, the odds ratio for CRP elevation was 3.7 (95% CI 2.1–6.5, seven studies) and for ESR elevation was 4.1 (95% CI 1.9–8.8, seven studies) in those with vascular activity on PET-CT when compared with those without [32]. Another systematic review reported that PET-CT had a sensitivity of 77% (95% CI 57–90%) and specificity of 71% (95% CI 47–87%) for diagnosing refractory or relapsing LVV [73].

Studies have also evaluated the role of PET-CT in predicting future relapses of LVV. In a study of 33 patients with clinically inactive TAK who were assessed by PET-CT, nine relapses were observed over a median of 4.5 years follow-up. After multivariable adjustment, an increased risk of relapse was predicted by the fulfillment of two points on the NIH score (hazard ratio 7.04, 95% CI 1.42–34.86) and by TBR >1.46 (hazard ratio 11.53, 95% CI 1.05–126.28) [74]. Some other studies have reported contrary results. In a study of 100 patients with LVV (49 with TAK) followed up for more than eight years, PETVAS could not accurately predict future relapses (hazard ratio 1.04, 95 percent CI 0.97–1.11) [70]. In another study of 32 patients with TAK (eleven of whom were active), SUV_max_ ≥ 1.3 at baseline was associated with greater odds of future relapse (OR 5.66, 95% CI 1.06–30.08) on univariable analyses but not after multivariable-adjusted analyses (hazard ratio for relapse 2.28, 95% CI 0.97–5.32) [75].

Limited data suggest that PET-CT might be useful to assess TAK with atypical features. From a study of 22 TAK patients diagnosed clinically but not fulfilling the 1990 American College of Rheumatology (ACR) criteria for TAK, PET-CT had 100% sensitivity and 75% specificity to detect active disease [76]. Pulmonary arteritis is a less frequent manifestation of TAK. In a study of 29 TAK patients with pulmonary artery involvement, PET-CT had higher diagnostic accuracy (84.2%) than CTA or MRA (57.9%) to detect active pulmonary arteritis. PET-CT uptake showed a moderate correlation with ESR or CRP. On follow-up imaging of eight patients, three had a decrease in uptake, four had similar uptake, and one had an increase in uptake on PET-CT [77].

PET-CT tagged with other ligands might have a role in assessing specific attributes of TAK. A recent paper reported PET-CT with Fibroblast Activator Protein Inhibitor (FAPI) tagged to gallium (FAPI-PET) in a young female with TAK. This patient had no uptake on 18-FDG PET but had florid aortic wall uptake on FAPI-PET [78]. There is a possibility that FAPI-PET might indicate areas of vascular fibrosis (thereby damage) in TAK; however, this needs to be explored further. Somatostatin receptor subtype 2 (SST2) PET might denote areas of macrophage infiltration in arteries and was reported to indicate active TAK [79].

Clinical trials of immunosuppressive therapies in TAK are few and far between. None of the high-quality trials have met their primary endpoint. A recent survey of international experts in TAK suggested the feasibility of using PET-CT to homogenize clinical trial recruitment in TAK [80].

The combination of PET with MR imaging for anatomical localization (PET-MRI) was explored in a few reports in TAK and GCA [81,82,83]. One reported greater uptake in PET-MRI with active LVV than inactive LVV [81]. Another study reported discordance between arterial wall uptake on PET-CT and vessel wall uptake on MRA [83]. The role of PET-MRI for the assessment of disease in TAK will require further evaluation.

**Figure 1:**
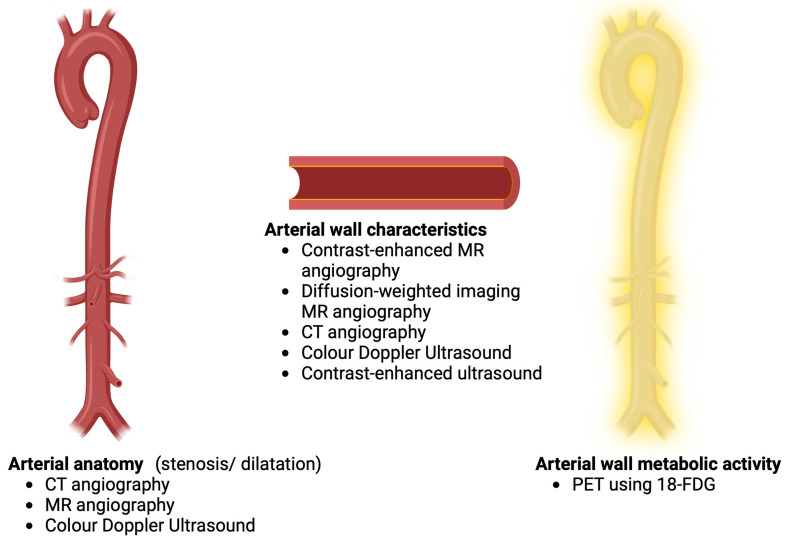
Utility of various imaging modalities for the assessment of the anatomy of the arterial tree, arterial wall characteristics, and metabolic activity of the vascular wall in TAK. Created with BioRender.com (Accessed on 17 October 2022). Abbreviations: 18-FDG—18-fluorodeoxyglucose; CT—Computerized tomography; MR—Magnetic resonance; PET—Positron emission tomography.

Figure 1 summarizes the various imaging modalities used for the assessment of the anatomy of the arterial tree, arterial wall characteristics, and metabolic activity of the vascular wall in TAK.

## 4. Circulating Biomarkers of Disease Activity in TAK

While various circulating biomarkers of disease activity have been described in TAK, fewer biomarkers have been evaluated to reflect vascular damage. These are summarized in Table 3 and detailed forthwith.

### 4.1. ESR

ESR is a ubiquitous, traditional biomarker of inflammation. However, ESR demonstrates an inconsistent association with disease activity in TAK. In a classical study from the NIH, ESR demonstrated a dissociation with disease activity. In total, 72% of those with active TAK and 56% with inactive TAK had elevated ESR. Four out of nine patients undergoing open surgical interventions for TAK had active vascular inflammation on histopathology despite being undertaken during periods of inactive disease (with a normal ESR) [84]. In another series of 33 patients with TAK from France undergoing vascular surgery, active inflammation (22%) and chronic inflammation (20%) were evident on histopathology despite clinically inactive disease (including ESR) at the time of surgery [85]. Thus, ESR does not reflect well disease activity in TAK.

### 4.2. Pentraxins

Pentraxins are a group of circulating inflammatory proteins. Short pentraxins are CRP and serum amyloid P (SAP), whose secretion from the liver is controlled by interleukin-6 (IL-6). Pentraxin-3 is a long pentraxin, secreted independently of IL-6 stimulation [86].

#### 4.2.1. C-Reactive Protein

CRP is a commonly used inflammatory marker. However, CRP inconsistently reflects disease activity in TAK. A study of 52 patients with TAK (15 serially before and after the initiation of immunosuppressive therapy) assessed quantitatively MRA to separately denote luminal narrowing, wall thickening, and wall enhancement. ESR (Spearman’s rho from 0.41–0.56), CRP (rho 0.32–0.61), and pentraxin-3 (rho 0.32–0.67) had a poor to moderate correlation with quantitative MRA scores. On serial follow-up, improvement in MRA wall enhancement scores was concurrent with decreases in ESR and CRP [42]. Another study of 54 patients with TAK assessed disease activity on PET-CT using semi-quantitative PETVAS scores and maximum standardized uptake values (SUV_max_) and its relationship with ESR, CRP, and pentraxin-3. Upon calculating the area under the curve (AUC) to distinguish active from inactive disease, pentraxin-3 (AUC 0.872) and PETVAS (AUC 0.856) had the highest distinguishing ability. AUC was lesser for SUV_max_ (0.771), ESR (0.686) and CRP (0.669). On longitudinal follow-up, PETVAS and pentraxin-3 demonstrated more consistent decreases with a concomitant reduction in disease activity than SUV_max_, ESR, or CRP [87]. A landmark paper assessed the relationship between vascular uptake on PET-CT and CRP in 30 patients with TAK. Sixteen of the 30 (46/177 vascular segments imaged) had increased 18-FDG uptake on PET-CT. Similar values of CRP were observed in TAK with and without active disease denoted on PET-CT. This suggested that PET-CT might identify a proportion of TAK with normal values of CRP despite active disease [72]. A systematic review with meta-analysis of six studies revealed a moderate effect size for the difference in CRP levels between those TAK with active or inactive disease on PET-CT (standardized mean difference 0.54, 95% confidence intervals—95% CI—0.15–0.92, I^2^ = 0%). Thus, CRP only moderately reflects TAK disease activity evident on a PET-CT [88]. From a study of 153 TAK who underwent serial CT angiography at a mean follow-up period of 3.53 years, 24 TAK showed angiographic progression. The AUC for CRP levels assessed longitudinally was associated with a greater risk of angiographic progression (hazard ratio 2.13, 95% CI 1.05–4.32) even after multivariable adjustment. ESR was associated with a higher risk of angiographic progression in univariable analyses but not in multivariable-adjusted analyses [89]. Another study longitudinally followed up 81 patients with TAK for up to three years. Of these, 59 attained remission, and 11 each either relapsed after attaining remission or had a treatment-refractory course. After multivariable adjustment, baseline CRP > 25 mg/L increased the odds of treatment refractoriness (odds ratio 1.61, 95% CI 1.21–20.64) but did not portend an increased risk of future relapses [90]. A study from the NIH, USA evaluated associations between various patient-reported outcome measures (PROMs, including patient global assessment of disease activity) with PGA of disease activity, CRP, ESR, and PETVAS scores in 56 patients each with TAK or GCA across 296 clinic visits. CRP was associated with active vasculitis even after multivariable-adjusted analyses. Both PGA (rho = 0.27) and patient global assessment of disease activity (rho = 0.16) were significantly associated with CRP, albeit the strength of association was poor [91]. Normalization of ESR or CRP is a potential measure indicating the control of active disease in TAK. However, overall few studies have reported this measure in observational studies or clinical trials of TAK [15,28]. One point to note is that tocilizumab, which is an interleukin-6 receptor antagonist, is one of the biologic DMARD used in TAK based on observational data and secondary endpoints met in an RCT [15,18]. In a patient receiving tocilizumab, the CRP levels are suppressed due to its action on IL-6 signaling and are no more reliable for assessing the disease activity of TAK.

The reason for the blunted CRP response in a proportion of TAK is unclear. Genetic variants in the promoter region of *CRP* are associated with lower or higher CRP levels. A study constructed eight haplotypes (H1 to H8) from seven different single nucleotide polymorphisms (SNPs) in the promoter region of *CRP* and assessed their relationship with CRP levels. The H1 haplotype was associated with lower levels of CRP than the H2 haplotype. The clade of H1 to H4 overall was associated with lower CRP levels than H5 to H8 [92]. We could only identify one study which had assessed the relationship of rs1205 SNP with TAK. In this study comparing 104 patients with TAK with 185 controls, the T allele was observed to be less frequently detected in TAK. The CC genotype was more common in TAK and did not associate with a blunted CRP response [93]. Future studies might consider exploring the role of genetic polymorphisms in *CRP* in determining blunted CRP response in a proportion of TAK.

#### 4.2.2. Pentraxin-3

The role of pentraxin-3 in distinguishing active vs. inactive TAK has been reported over the past decade. In a study of 41 TAK patients (23 with active disease), serum pentraxin-3 was more sensitive (82.6% vs. 65.2%) and less specific (77.8% vs. 94.4%) with overall better performance than CRP (AUC 0.914 vs. 0.905) to distinguish active TAK. Pentraxin-3 was elevated in six of the eight patients with active TAK who had undetectable CRP levels. Levels of pentraxin-3 did not correlate with the dose of prednisolone. Immunohistochemistry of aortic tissue from TAK revealed staining for PTX-3 on inflammatory cells and on the vasa vasorum (endothelium) [94]. Another study of 57 patients with TAK (27 with active disease) revealed higher plasma pentraxin-3 levels in active TAK than in those with inactive disease. Similar observations also held true for ESR and CRP. However, pentraxin-3 had better performance to distinguish active TAK from inactive disease (AUC 0.919, 95% CI 0.847–0.991) than ESR (AUC 0.750, 95% CI 0.623–0.876) or CRP (AUC 0.684, 95% CI 0.582–0.850) [95]. A study of 35 patients with TAK evaluated circulating pro-angiogenic and anti-angiogenic factors. The pro-angiogenic vascular endothelial growth factor (VEGF) and anti-angiogenic pentraxin-3 (but not other factors) were elevated in TAK than in healthy controls. Both VEGF and pentraxin-3 were associated with active vascular disease on PET-CT in a proportion of patients with TAK [96]. Another study of 42 patients with TAK evaluated pentraxin-3 as a marker of disease activity or vascular involvement. Plasma levels of plasma pentraxin-3 and CRP were similar in TAK who were active (12/42) or inactive by the NIH criteria, and in TAK with angiographic progression on serial imaging (9/40) when compared with those without. Those TAK with vascular enhancement on CTA or MRA (5/30) had higher pentraxin-3 but similar CRP levels to those without. In a subset of patients who were not receiving anti-cytokine biologic disease-modifying anti-rheumatic drugs (DMARDs), pentraxin-3 was higher in those with vascular enhancement or those with angiographic progression than without. CRP was similar in both groups. ESR was elevated in those with vascular progression than those without, but similar in those with or without vascular wall enhancement [97]. In another study of 98 patients with TAK, those with active disease as per NIH criteria (45/98) or as per the criteria used in the abatacept trials in LVV (52/98) had higher serum levels of pentraxin-3 when compared to inactive TAK by either of the criteria. Serum levels of lysosomal-associated membrane protein 2 (implicated in the pathogenesis of AAV) were similar in TAK with active or inactive disease [98]. In a small cohort of 17 patients with TAK, plasma pentraxin-3 (but not other cytokines, chemokines, and growth factors) was associated with NIH disease activity scores. Furthermore, the levels of pentraxin-3 reduced with a concomitant decrease in NIH scores following treatment with tofacitinib and glucocorticoids [99]. However, not all studies have reported an association of pentraxin-3 with active TAK. In a study of 94 patients with TAK (33/94 active as per physician global assessment, 25/94 active as per NIH criteria, and 28/94 active as per ITAS2010 greater than 1 or ITAS-A greater than 4), the plasma levels of pentraxin-3 were similar in active or inactive TAK [100]. A systematic review with meta-analysis of 473 patients with TAK (208 with active disease) from eight studies reported a moderate effect size for differences in levels of circulating pentraxin-3 between active and inactive TAK (standardized mean difference 0.761, 95% CI 0.300–1.140, I^2^ 68%). Further, from five of these studies which reported both pentraxin-3 and CRP, the pooled sensitivity (78% vs. 66%), specificity (85% vs. 77%) and AUC (0.88, 95% CI 0.85–0.90 vs. 0.75, 95% CI 0.71–0.79) were better for pentraxin-3 than for CRP [101]. Some studies [100], but not others [97], have demonstrated a correlation between circulating levels of CRP and pentraxin-3 in TAK. Limited data suggest that pentraxin-3 may associate with specific manifestations of TAK. A study compared 51 patients with different vasculitides (including seven with TAK) with 104 healthy controls or controls with essential hypertension. Similar levels of pentraxin-3 were observed between active and inactive vasculitis. However, vasculitis with hypertension had higher levels of pentraxin-3 than those with essential hypertension or healthy subjects [102].

### 4.3. Biomarkers Derived from Routine Hemogram Reports

Total and differential leukocyte counts and platelet counts are routinely carried out for monitoring most patients with autoimmune diseases, including TAK. A study evaluated the platelet-to-lymphocyte ratio (PLR) and neutrophil-to-lymphocyte ratio (NLR) as biomarkers of disease activity in 88 patients with TAK (43 of whom had active disease). Both PLR and NLR were increased in active TAK when compared with inactive TAK. When compared against the NIH score, PLR (AUC 0.691, 95% CI 0.580–0.802) and NLR (AUC 0.697, 95% CI 0.588–0.806) had moderate performance to distinguish active TAK [103]. Another study compared NLR, PLR, monocyte-to-lymphocyte ratio (MLR), and CRP: albumin ratio in 32 patients with active TAK with paired values after attaining remission. All these parameters were increased in active TAK than during remission. CRP: albumin ratio had the highest AUC (0.999), followed by NLR (0.869), MLR (0.677) and PLR (0.652) to distinguish active from inactive TAK [104].

The red cell distribution width (RDW) is another parameter reported by most automated cell counters used to assess hemograms. Increased RDW is associated with inflammation. In a study of 156 TAK (69 of whom had active disease), RDW was increased in active TAK when compared with inactive TAK. Levels of CRP associated with RDW after multivariable-adjusted analyses [105].

### 4.4. Cell Populations in the Peripheral Blood and Their Related Trophic Factors

Inflammatory cells play a key role in the pathogenesis of TAK. Infiltration of lymphocytes was demonstrated in the histopathology of arteries involved in TAK [3]. Over the past decade, the role of circulating lymphocyte populations in driving TAK disease has become clearer. A study explored circulating T helper (Th) 1 and Th17 lymphocytes in 41 patients with TAK (17 with active disease). Increased circulating Th1 and Th17 lymphocytes were detected in active TAK than in inactive TAK. Naïve T lymphocytes were fewer in active TAK. No differences were observed in central or effector memory T lymphocytes in relation to the disease activity of TAK. Th1 cytokines but not Th17 cytokines were suppressed following glucocorticoid therapy in TAK [106]. Another study of 30 patients with TAK revealed similar proportions of circulating Th17 lymphocytes and serum IL-17 and IL-23 in patients with active (denoted by NIH criteria (n = 12) or ITAS2010 ≥ 4 (n = 13)) or inactive TAK [107]. Over the past decade, a population of T lymphocytes (Th17.1 lymphocytes) with the ability to secrete both interferon-gamma (such as Th1 lymphocytes) and IL-17 (such as Th17 lymphocytes) was identified. Some studies have suggested that such Th17.1 lymphocytes might be resistant to corticosteroids, as they express the drug efflux protein p-glycoprotein [108]. A recent study comparing 30 patients with active TAK with 27 patients with inactive TAK reported increased Th17 and Th17.1 lymphocytes in active TAK on univariable analyses. However, Th17 lymphocytes (but not Th17.1 lymphocytes) remained associated with active TAK after adjustment for CRP and Th1, Th2, and T regulatory lymphocyte populations [109]. Another study analyzed gene expression on peripheral blood mononuclear cells in eleven patients with active TAK compared with nine with inactive TAK (disease activity assessed using the NIH criteria). Active TAK had increased expression of genes related to the T-cell receptor, T lymphocyte proliferation (*CD28*), Th2 lymphocytes (*GATA3)*, and Th17 lymphocytes (*RORC*) and decreased expression of *CD40* (which down-regulates T lymphocyte activation). TAK with active disease had a clustering of genes related to the T-cell receptor activation when compared with inactive TAK. Such clustering could distinguish active TAK very well with an AUC of 0.98 [110].

Based on the comparison of immunohistochemistry of involved arteries in TAK, GCA, and non-LVV control subjects, a study postulated the role of the mammalian target of rapamycin complex 1 (mTORC1) in the pathogenesis of LVV. Further, when in vitro cultured peripheral blood mononuclear cells were treated with rapamycin (which inhibits mTORC1), a decreased secretion of interferon-gamma, IL-17, and IL-21 in the culture supernatant was observed in TAK, GCA and control subjects. However, the proportion of CD4+ T lymphocytes in the culture secreting each interferon-gamma (Th1), IL-17 (Th17), and IL-21 were decreased following treatment with rapamycin in TAK but not in GCA or controls. This suggested the potential role of the Th1, Th17, and T-lymphocytes secreting IL-21 as biomarkers of active TAK [111].

Infiltration of macrophages was also described in the histopathology of TAK [3]. A recent study described macrophage subsets in TAK. Fifteen patients with TAK (eight untreated) had histopathological tissue available for analysis. Increased M1 macrophages were demonstrable in the vessel wall of untreated TAK in the adventitial layer, concomitant with increased expression of the chemokine (C-C motif) ligand 2 (CCL2) in the same location. Conversely, in those patients with TAK on immunosuppressive treatment, the expression of CCL2 in the arterial wall was much reduced, and mostly present in the media layer (where infiltration of M2 macrophages was dominant). Further, the authors evaluated serum CCL2 levels in 59 patients with TAK (46 of whom had active disease as per the NIH criteria). Serum CCL2 levels were elevated in active TAK than in inactive TAK. Serum CCL2 as a predictor of active TAK (AUC 0.74) had a similar performance to CRP (AUC 0.83) but an inferior performance to ESR (AUC 0.93) [112]. In this context, it is important to reiterate that ESR is a component of the NIH disease activity criteria.

Unlike in other autoimmune diseases such as systemic lupus erythematosus, B lymphocytes are scarcely implicated in the pathogenesis of TAK. Circulating early plasmablasts (which secrete antibodies) were found to be elevated in TAK when compared with healthy controls. The same paper also reported the successful amelioration of disease activity with rituximab (which depletes B lymphocytes, which are the source of plasmablasts) in three TAK patients with a refractory disease course [113]. Another study reported serum levels of B-cell activating factor (BAFF) and a proliferation-inducing ligand (APRIL), both of which drive B lymphocyte proliferation and differentiation, in 50 patients with TAK (24 with active disease). BAFF was similar in active and inactive TAK, whereas APRIL was elevated in active TAK on univariable analyses. Neither BAFF nor APRIL correlated with ITAS-A calculated using ESR or with damage denoted by the TADS [114]. Subsequent reports have not shown a beneficial effect of rituximab on TAK, thereby raising doubts about whether B lymphocytes play a significant role in TAK pathogenesis or disease activity [15,115,116].

### 4.5. Cytokines

Inflammatory cells infiltrating the aortic wall secrete several inflammatory cytokines such as tumor necrosis factor-alpha (TNF-α) and IL-6. Therefore, it was hypothesized that inflammatory cytokines in the peripheral blood might associate with the disease activity of TAK. A study of 53 patients with TAK (20 with active disease) reported higher levels of serum IL-8 in active TAK when compared with inactive TAK. In a subset of TAK with active disease at baseline, sequential samples showed normalization of IL-8 levels concomitant with the attainment of clinical remission [117]. Another study of 51 patients with TAK (21 with active disease by the NIH criteria) revealed higher levels of IL-8, IL-23, and IL-10 in those with the inactive disease. Furthermore, when the same authors compared cytokine levels in those eight TAK active as per the ITAS2010 score with the others, the serum levels of IL-18 were higher in active TAK [118]. Serum IL-6 was found to moderately correlate with ESR, CRP, and NIH scores in a cross-sectional study of 50 patients with TAK [119]. In another study of 14 patients with active TAK when compared with 11 with inactive TAK, the levels of serum IL-6 (but not other inflammatory cytokines) were higher in active TAK [120]. A large study of 488 patients with TAK (188 with active disease) assessed the relationship of ESR, CRP, serum IL-6 and TNF-α with the risk of future remission in those with active disease at baseline, and relapses in those with the inactive disease at baseline. In those with active disease at baseline, higher levels of ESR, CRP, and IL-6 were associated with a lower risk of remission and with a longer time to remission. In those with inactive disease at baseline, higher levels of ESR, CRP, IL-6, and TNF-α each individually predicted a greater risk of relapse on follow-up as well as earlier relapse [121]. Another study of 67 patients with TAK (56 with active TAK) assessed the relationship between baseline serum IL-6 levels and disease course over a follow-up period of at least two years. Serum IL-6 at baseline was higher in TAK with active disease than with inactive disease. IL-6 moderately correlated with CRP and was higher with an increasing number of items on the NIH disease activity score. After categorizing baseline IL-6 into three groups of low, moderate, and high levels (cut-offs of 40^th^ percentile and 70^th^ percentile), a dose–response association between baseline IL-6 and future relapses was not evident. After multivariable-adjusted Cox regression analyses, the risk of relapse was significantly higher with moderate levels of IL-6 at baseline (hazard ratio 4.3, 95% CI 1.3–18.7) but not with high baseline levels of IL-6 (hazard ratio 2.1, 95% CI 0.7–48.9) [122]. Some studies have also shown contrary results related to IL-6 in TAK. A study of 26 TAK with active disease compared with eighteen with inactive disease reported similar levels of IL-6 in both groups [123]. A further study compared serum cytokines in 29 TAK with active disease with 27 with inactive disease. The levels of inflammatory or regulatory cytokines were not significantly different between these two groups [109]. Another study compared various inflammatory and regulatory cytokines (including IL-6) between 15 TAK with the active disease and 17 with inactive disease. Interferon-gamma was increased, but other cytokines (including IL-6) were not significantly different between TAK with active or inactive disease [93]. When peripheral blood mononuclear cells isolated from healthy individuals were treated in vitro with sera from patients with active TAK, the cytokines interferon-gamma and IL-17 were both secreted in greater amounts. This suggests the role of interferon-gamma and IL-17 in active TAK [106].

Targeting IL-6 with the IL-6 receptor antagonist tocilizumab in TAK was explored in TAK. In a phase 3 RCT comparing 18 TAK patients each treated with tocilizumab or placebo, the time to relapse was similar between tocilizumab and placebo in the intention-to-treat primary analysis but favored tocilizumab in the per-protocol secondary analysis [18]. On a longer-term follow-up on these patients, including an open-label extension phase, 60% of TAK treated with tocilizumab experienced a stabilization of angiographic progression [124]. Observational data also support the use of tocilizumab in TAK [15]. On the basis of these studies, tocilizumab is one of the therapeutic options for treatment-refractory TAK [17]. Targeting of TNF-α has also resulted in favorable outcomes in TAK based on observational data [15]. A recent multicentric cohort study reported similar outcomes in TAK treated with tocilizumab or TNF-α inhibitors [125].

### 4.6. Autoantibodies

Few studies have reported autoantibodies in TAK. A study of 30 TAK patients (13 with active disease) identified circulating anticardiolipin antibodies and anti-endothelial cell antibodies more frequently in active TAK when compared to those with inactive disease. In a third of patients, both anticardiolipin and anti-endothelial cell antibodies were coexistent [126]. In another study of 66 TAK (of whom 36 had active disease), anti-annexin V antibodies were elevated in active TAK when compared with inactive TAK. Out of 12 patients with active TAK for whom longitudinal samples were available, the levels of anti-annexin V antibodies normalized concurrently with reductions in disease activity [127].

### 4.7. Markers of Endothelial Injury

Endothelial injury is one of the pathogenic mechanisms operating in TAK. Circulating endothelial progenitor cells (EPCs) were elevated in TAK when compared with Behcet’s disease or with healthy controls. The levels of circulating EPCs moderately correlated with ESR and CRP [128]. A study of 35 patients with TAK (20 with active disease) reported similar levels of soluble E-selectin, vascular cell adhesion molecule 1 (VCAM-1), and intercellular adhesion molecule 1 (ICAM-1) in active or inactive TAK. The levels of these biomarkers remained similar before and after immunosuppression in fifteen patients over a period of 12 months [129]. Another study of 32 patients with TAK (of whom seven had active disease) reported similar levels of circulating endothelial cells (CECs) but higher levels of circulating EPCs and VEGF in active TAK when compared with inactive TAK. Circulating EPCs and VEGF also demonstrated a weak to moderate correlation with CRP and ITAS-A calculated using CRP [130].

### 4.8. Matrix Metalloproteinases

The role of matrix metalloproteinases (MMPs) as biomarkers of disease activity in TAK was first assessed in 25 patients (11 of whom had active disease). Serum levels of MMP-3 and MMP-9 were increased in active TAK when compared with inactive TAK and decreased when measured serially as active TAK went into remission. The levels of MMP-2 and tissue inhibitor of metalloproteinases 1 (TIMP-1) were similar between active and inactive TAK [131]. Another study of 40 TAK (28 active) revealed higher serum levels of MMP-2 and MMP-9 in active TAK versus inactive TAK on univariable analyses but not after multivariable adjustment for other cytokines [132]. In another report of 40 patients with TAK, of whom 32 had active disease, the gelatinolytic activity of MMP-2 and MMP-9 was higher in active than in inactive TAK on univariable analyses [133]. Some studies have reported contrary results. In 44 TAK patients (26 with active disease), MMP-9 was increased and MMP-3 was decreased in active TAK than in inactive TAK. MMP-9 had an AUC of 0.97 (95% CI 0.93–1.00) to distinguish active from inactive TAK. The addition of MMP-9 to CRP increased the sensitivity (93.08%) and specificity (96.30%) to detect active disease when compared with CRP alone (sensitivity 53.86%, specificity 72.22%) [123]. Another study reported similar serum levels of MMP-3 and MMP-9 in 14 patients with active TAK when compared with another 11 with inactive TAK [120]. A study comparing 23 active TAK with another 18 with inactive disease also reported similar serum levels of MMP-2 and MMP-3 between both groups [94].

### 4.9. Other Biomarkers of Disease Activity in TAK

Serum amyloid A (SAA) was reported to be increased in 43 patients with active TAK when compared with 56 others with inactive TAK. SAA had a similar performance (AUC 0.877, 95% CI 0.808–0.946) to CRP (AUC 0.858) and better performance than ESR (AUC 0.645) to distinguish active TAK. On serial follow-up, the levels of SAA decreased in those who had a reduction in disease activity (concomitant with a reduction in CRP) when compared with non-responders [134].

S100 proteins are disease-associated molecular patterns that are intricately linked with immune responses. Serum levels of a dimer of S100A8/S100A9, also called calprotectin or myeloid-related protein 8/14 (MRP 8/14), were evaluated in 85 TAK (55 with active disease). MRP 8/14 was elevated in active than in inactive TAK. On serial follow-up, those with a demonstrable clinical response of disease activity showed reductions in serum MRP 8/14. Such a reduction was not evident in non-responders or those with relapses of TAK. A greater proportion (66%) of those with the angiographic progression of TAK had increases in MRP 8/14 on serial follow-up when compared with those not showing angiographic progression (26%) [135]. Other studies have shown contrary results. In a study of 16 patients with TAK, serum MRP8/14 and S100A12 were similar in active or inactive disease [136]. Another study of 29 patients with TAK (11 with active disease) reported higher levels of fecal S100A12 in active TAK. Fecal S100A12 levels demonstrated a significant moderate correlation with ITAS2010 but not with ESR or CRP [137].

Serum complement fraction 1q (C1q) was evaluated as a biomarker in 198 patients with TAK (47 with active disease). Those with active disease had higher levels of serum C1q than those with inactive disease. Serum C1q levels demonstrated a moderate correlation with NIH disease activity scores, ESR, and CRP. However, serum C1q levels performed no better (AUC 0.752) than ESR (AUC 0.825) or CRP (AUC 0.834) to distinguish active from inactive TAK. Combining information from all three C1q, ESR and CRP did not significantly improve the prediction of active TAK (AUC 0.845) [138]. Serum levels of complement component 3 (C3) are known to be elevated in systemic vasculitis. A recent study reported elevated serum C3 levels in 406 patients with active TAK when compared with 113 with inactive TAK. Elevated serum C3 levels had moderate performance to distinguish active from inactive TAK (AUC 0.715, 95% CI 0.650–0.781), similar to ESR and CRP, without significant change in AUC when ESR or CRP was incorporated with serum C3 [139].

Higher baseline levels of serum leptin, a pro-inflammatory adipokine, were associated with progression on serial angiography in a cohort of 34 TAK patients followed up over five years. Those with the highest tertile of baseline leptin levels had a significantly increased hazard of angiographic progression when compared with the lowest tertile (hazard ratio 12.68, 95% CI 1.06–152.44) after adjustment for age of disease onset, duration of disease, and baseline disease activity assessed using the NIH score [140].

Fetuin-A is a negative circulating acute phase reactant. A study of 32 patients with TAK (14 with active TAK) reported a weak negative correlation of serum fetuin-A with CRP or ITAS2010. Serum fetuin-A less than 66.2 ng/mL had a good performance to distinguish active from inactive TAK (AUC 0.858) [141].

### 4.10. Circulating Proteomic Signatures

Proteomics is a hypothesis-free technique to screen circulating proteins in plasma or serum that might distinguish diseases or disease activity states. In a study of 43 patients with TAK (18 with active disease), the authors identified eleven plasma proteins that were distinct between TAK and healthy controls by proteomics screening in a previous study. Of these, they reported serum amyloid A and C4 binding protein (C4BP) to be increased in active TAK when compared with inactive TAK [142]. In another study, after proteomics screening using protein microarrays of plasma from 60 patients with TAK (29 with active disease) and 30 healthy controls, eight proteins were identified as significantly different in active and inactive TAK (CA125, FLRG, IGFBP-2, CA15-3, GROα, LYVE-1, ULBP-2, and CD99). The combined AUC for these eight proteins to distinguish active TAK from inactive TAK was 0.909 (sensitivity 81.82%, specificity 100%) [143]. From a study of 80 patients with TAK (40 of whom had active disease) and 40 healthy controls, plasma proteins were distinguished between TAK and controls using 2D gel electrophoresis. Differently expressed protein spots were further identified using mass spectroscopy. Three proteins (SAA, C4BP, and RAG-1) were significantly different between active and inactive TAK. The composite performance of these three proteins to distinguish active vs. inactive TAK (AUC 0.94, 95% CI 0.86–0.98) was better than either ESR (AUC 0.71, 95% CI 0.60–0.80) or CRP (AUC 0.75, 95% CI 0.64–0.84) [144]. A recent paper analyzed serum proteomics using protein microarray in TAK vs. healthy controls. Although eight proteins could distinguish TAK from healthy controls, none of these were significantly different between 31 patients with active TAK and 78 with inactive TAK [145]. Another study screened the sera of five patients with TAK and three healthy controls using a chemokine array and identified twelve chemokines to be increased and four to be decreased in TAK than in controls. Further validating these chemokines in 20 TAK patients and 20 healthy controls, five chemokines (CCL22, RANTES, CXCL11, CXCL16, IL-16) were confirmed to be elevated in TAK. However, none of these cytokines were associated with ESR or CRP or were significantly different between active and inactive TAK in a separate cohort of 25 patients with TAK [146].

### 4.11. Serum Metabolomic Signatures

Serum metabolomic signatures are increasingly being recognized in immune-mediated inflammatory diseases including TAK. A study compared serum metabolomic signatures in 98 patients with TAK (45 with active disease indicated by ITAS-A ≥ 4) as markers of active TAK. Increased glutamate and proline best distinguished active TAK from inactive TAK (AUC 0.816, 95% CI 0.721–0.891), followed by glutamate alone (AUC 0.775, 95% CI 0.685–0.859) and N-acetyl glycoprotein (AUC 0.769, 95% CI 0.675–0.847). Other metabolites which could distinguish active vs. inactive TAK were increased glucose (AUC 0.760, 95% CI 0.653–0.851), glycerol (AUC 0.746, 95% CI 0.645–0.850), phosphoglyceride (AUC 0.743, 95% CI 0.643–0.834), phenylalanine (AUC 0.720, 95% CI 0.611–0.816) and decreased low-density lipoprotein cholesterol (AUC 0.719, 95% CI 0.615–0.807) [147]. From the same cohort, a decrease in the circulating glutamine to glucose ratio (AUC 0.758, 95% CI 0.658–0.839) and lactate to glucose ratio (AUC 0.667, 95% CI 0.561–0.768) could also distinguish active from inactive TAK better than ESR (AUC 0.671, 95% CI 0.561–0.770) or CRP (AUC 0.649, 95% CI 0.539–0.749) [148].

### 4.12. Circulating Markers of Vascular Damage

Most studies have focused on distinguishing the disease activity of TAK based on circulating biomarkers or imaging modalities. Inflammation in TAK results in vascular fibrosis, which is clinically manifested as vascular stenosis. A recent study evaluated the relationship of an established circulating biomarker of liver fibrosis, the enhanced liver fibrosis (ELF) score, with vascular damage in TAK. The ELF score comprises three serum markers, TIMP-1, hyaluronic acid, and amino-terminal peptide of procollagen type III. Twenty-four patients with TAK were included in this study. The overall ELF score and individually hyaluronic acid and amino-terminal peptide of procollagen type III moderately correlated with the VDI, TADS, and with the number of vascular regions involved, and strongly correlated with the combined arteritis damage score (CARDS). TIMP-1 only moderately correlated with the number of vascular regions involved. Since ELF was also associated significantly with age, after adjusting the association between ELF and CARDS for age, the magnitude of the correlation remained strong (Pearson’s r = 0.73) [149].

Based on genome-wide association studies (GWAS) from Japan comparing patients with TAK and healthy controls, a risk allele (A) in the *IL12B* region (rs6871626, which encodes for the IL12p40 subunit) was associated with susceptibility to TAK [150,151]. On functionally characterizing the rs6871626 single nucleotide polymorphism in 44 patients with TAK, those bearing the risk allele had higher plasma levels of IL12p40 and IL-12p70 and increased levels of IL-12p70 in the supernatant of ex vivo cultured monocytes from TAK patients. The presence of this risk allele is also associated with higher circulating proportions of Th1 (but not Th17) lymphocytes [152]. Further, in 99 patients with TAK, homozygosity or heterozygosity for the A allele of rs6871626 was associated with higher damage scores indicated by the TADS and the VDI [153].

**Figure 2:**
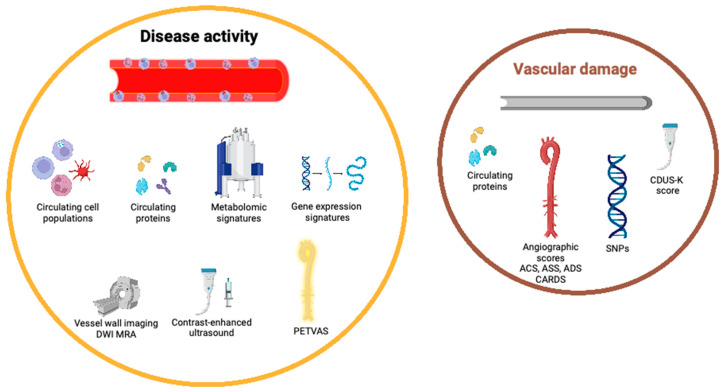
Biomarkers for disease activity and vascular damage in Takayasu arteritis. Created with BioRender.com (Accessed on 17 October 2022). Abbreviations: ACS—Angiographic Composite Score; ADS—Angiographic Dilatation Score; ASS—Angiographic Stenosis Score; CARDS—Combined Arteritis Damage Score; CDUS-K—Color Doppler ultrasound in Takayasu arteritis score from Kolkata; DWI—Diffusion Weighted Imaging; MRA—Magnetic resonance imaging; PETVAS—Positron emission tomography vascular activity score; SNPs—Single nucleotide polymorphisms.

Figure 2 summarizes the various biomarkers of disease activity and vascular damage in TAK.

## 5. Composite Scoring Systems Involving Clinical Assessment, Radiologic Findings, and Circulating Biomarkers

It is evident that the assessment of disease activity and vascular damage in TAK is challenging. Combining information available from diverse modalities may enable a holistic assessment of disease activity in TAK [37]. A recent study evaluated the combination of information from ESR, CRP, IL-6, soluble IL-2 receptor (sIL-2R), and PET-CT to assess disease activity in 91 patients with TAK (65 of whom had active disease. The performance of the models to distinguish active from inactive TAK was assessed by estimating C-index for the area under receiver operating characteristics curves. The C-index for ESR alone was 0.78 (95% CI 0.69–0.88). Individually, CRP, IL-6, IL-2R, or individual parameters derived from PET-CT, i.e., the total maximum value of standardized uptake value (SUVmax) or total mean value of SUV (SUVmean), had a similar or worse C-index than ESR alone. Combinations of ESR with total SUVmax/total SUVmean sIL-2R/IL-6 were evaluated against ESR alone for assessing active disease. A model including information from ESR, total SUVmean, and sIL-2R had the maximum C-index (0.96, 95% CI 0.94–1.00) and significantly better performance than ESR alone to delineate active TAK. This model was also superior to the NIH score alone (AUC 0.87) to identify active TAK [154]. This study provided an excellent approach to summating information from different parameters (traditional inflammatory markers, circulating soluble proteins, and metabolic activity evident on PET-CT) to distinguish active vs. inactive TAK. However, this model would require validation in different populations before it could be directly applied to assess disease activity in TAK.

## 6. Future Perspectives

The proposed agenda for future research related to outcome measures and biomarkers are delineated in Table 4. There remains an unmet need to modify the DEI.TAK and ITAS2010 to incorporate information from imaging. Validated cut-offs for active disease using the ITAS2010 and DEI.TAK also need to be established. The lack of a validated damage index for TAK is another agenda for future research. Such a damage index needs to be data-driven rather than being adapted from indices such as the VDI, which are meant for use primarily in small or medium vessel vasculitis. A TAK-specific PROM which includes information as deemed relevant by patients with TAK from different geographic regions, as well as accounts for disease features of TAK, is also an agenda for future research. Development and validation of composite scoring systems incorporating information from imaging, circulating biomarkers, and clinical indices might enable the robust assessment of disease activity in TAK in the future.

## Figures and Tables

**Table 1:** Items common to TADS, DEI.TAK, and ITAS2010.

Items Common to the DEI.TAK and TADS		Items Common to the ITAS2010 and TADS	
Domain	Items	Domain	Items
Renal	Systolic hypertension * Diastolic hypertension * Proteinuria Elevation of serum creatinine	Renal	Systolic hypertension * Diastolic hypertension *
Nervous system	Organic confusion/dementia Seizures Stroke Cord lesion	Nervous system	Stroke Seizures
Cardiovascular system	Vascular bruits Pulse and BP inequality Pulse loss Limb claudication Aortic regurgitation Ischemic cardiac pain Congestive cardiac failure Cardiomyopathy	Cardiovascular system	Vascular bruits Pulse and BP inequality Pulse loss Limb claudication Aortic regurgitation Ischemic cardiac pain Congestive cardiac failure Cardiomyopathy
Eyes	Vision loss	-	-
Chest	Persistent cough/wheeze/dyspnea	-	-

* Different cut-offs were used for systolic and diastolic hypertension in the DEI.TAK/ITAS2010 and in the TADS.

**Table 2:** Measures for the assessment of disease activity and damage in TAK.

Domain	Scoring System or Criteria
Disease activity	
Clinical	National Institutes of Health (NIH) disease activity score
	Disease Extent Index in Takayasu Arteritis (DEI.TAK)
	Indian Takayasu Clinical Activity Score (ITAS2010) and ITAS2010 modified for acute phase reactants ESR or CRP (ITAS-A)
	Abatacept in Giant Cell Arteritis and Takayasu arteritis (AGATA) criteria
	Dabague criteria
	European Alliance of Associations for Rheumatology (EULAR) criteria
	Future relapses
	Time-to-relapse
	Percentage reduction in corticosteroid dose from baseline
Imaging	Positron Emission Tomography Vasculitis Activity Score (PETVAS)
**Damage**	
Clinical	Vasculitis Damage Index (VDI)
	Combined Damage Assessment Score (CDA)
	Takayasu Arteritis Damage Score (TADS)
Imaging	Color Doppler Ultrasound Score from Kolkata (CDUS-K) *
	Angiographic Stenosis Score (ASS), Angiographic Dilatation Score (ADS) and Angiographic Composite Score (ACS)
	Combined Arteritis Damage Score (CARDS)

* Although the CDUS-K was reported as an activity score, it likely better reflects vascular damage.

**Table 3:** Circulating biomarkers for disease activity and damage in TAK.

Domain	Biomarker
Disease activity	
Acute phase reactants	ESR
	CRP
	Pentraxin-3
Biomarkers from routine hemogram	Platelet-to-lymphocyte ratio
	Neutrophil-to-lymphocyte ratio
	Monocyte-to-lymphocyte ratio
	Red cell distribution width
Cell populations in peripheral blood and their related trophic factors	Th1, Th17 and Th17.1 lymphocytes
	Gene expression of *TCR*, *CD28*, *GATA3, RORC* (increased expression) and *CD40* (decreased expression)
	CCL-2
	BAFF, APRIL
Cytokines	IL-6, TNF-α, IL- 8, IL- 23, IL-10, IL-18
Autoantibodies	Anti-cardiolipin antibodies
	Anti-endothelial cell antibodies
	Anti-annexin V
Markers of endothelial injury	Circulating ECs and EPCs
	VCAM-1, ICAM-1, VEGF
Matrix metalloproteinases	MMP-2, MMP-3, MMP-9, TIMP-1
Miscellaneous biomarkers	SAA
	S100A8/S100A9/MRP 8/14
	S100A12
	C1q
	C3
	Leptin
	Fetuin-A
Proteomics	Serum amyloid A, C4BP, RAG-1
	CA125, FLRG, IGFBP-2, CA15-3, GROα, LYVE-1, ULB-2, CD99
	CCL-22, RANTES, CXCL-11, CXCL-16, IL-16
Metabolomics	Glutamate, proline, N-acetyl glycoprotein, glucose, glycerol, phosphoglyceride, phenylalanine, low-density lipoprotein cholesterol
	Glutamine-to-glucose ratio; Lactate-to-glucose ratio
**Vascular damage**	
ELF score	TIMP-1, hyaluronic acid, amino-terminal peptide of procollagen type III

APRIL—A proliferation-inducing ligand; BAFF—B-cell activating factor; C1q—complement fraction 1q; C3—Complement component 3; CCL-2—chemokine (C-C motif) ligand 2; CRP—C-reactive protein; EC—Endothelial cells; ELF—enhanced liver fibrosis; EPC—Endothelial progenitor cells; ESR—Erythrocyte sedimentation rate; ICAM-1—Intercellular adhesion molecule 1; IL- Interleukin; MMP—Matrix metalloproteinases; MRP-8/14—myeloid-related protein 8/14; TIMP-1—Tissue inhibitor of metalloproteinases 1; SAA—Serum amyloid A; Th—T helper; TNF-α—Tumor necrosis factor-alpha; VCAM-1—Vascular cell adhesion molecule 1; VEGF—Vascular endothelial growth factor.

**Table 4:** Agenda for future research related to outcome measures and biomarkers for TAK.

Delineation of validated cut-offs for DEI.TAK and ITAS2010.
Modification of DEI.TAK and ITAS2010 to incorporate information from imaging.
Development and validation of new disease activity scoring systems using a data-driven approach.
Development and validation of damage indices for TAK using a data-driven approach.
Development of a TAK-specific patient-reported outcome measure.
Development and validation of composite scoring systems incorporating information from clinical disease activity scores, vascular imaging, and circulating biomarkers.
Identification of biomarkers to reflect vascular damage in TAK.

DEI.TAK—Disease extent index in TAK; ITAS2010—Indian Takayasu arteritis Clinical Activity Score; TAK—Takayasu arteritis.

## Data Availability

Not applicable.

## Abbreviations

AAV	ANCA-associated vasculitis
ACS	Angiographic Composite Score
ADS	Angiographic Dilatation Score
AGATA	Abatacept in Giant Cell Arteritis and Takayasu arteritis
ANCA	Antineutrophil cytoplasmic antibody
APRs	Acute phase reactants
ASS	Angiographic Stenosis Score
AUC	Area under the curve
BVAS	Birmingham Vasculitis Activity Score
CA125	Cancer antigen 125
CA15-3	Cancer antigen 15-3
CARDS	Combined Arteritis Damage Score
CCL	Chemokine (C-C motif) ligand
CDUS	Colour Doppler ultrasound
CDUS-K	Colour Doppler ultrasound in Takayasu arteritis score from Kolkata
CDA	Combined Damage Assessment Index
CECs	Circulating endothelial cells
CEMRA	Contrast-enhanced MRA
CRP	C-reactive protein
CTA	Computerized tomographic angiography
C4BP	C4 binding protein
DEI.TAK	Disease Extent Index in TAK
DMARDs	Disease-modifying anti-rheumatic drugs
DSA	Digital subtraction angiography
EPCs	Endothelial progenitor cells
ESR	Erythrocyte sedimentation rate
EULAR	European Alliance of Associations for Rheumatology
FAPI	Fibroblast Activator Protein Inhibitor
18-FDG	18-fluorodeoxyglucose
GCA	Giant cell arteritis
GFR	Glomerular filtration rate
GROα	Growth-regulated oncogene α
IGFBP-2	Insulin like growth factor binding protein 2
IL	Interleukin
ICAM-1	Intercellular adhesion molecule 1
ITAS	Indian TAK Clinical Activity Score
LVV	Large vessel vasculitis
LYVE-1	Lymphatic vessel endothelial hyaluronan receptor 1
MRA	Magnetic resonance angiography
MMPs	Matrix metalloproteinases
MRP 8/14	Myeloid-related protein 8/14
NLR	Neutrophil-to-lymphocyte ratio
PAAT	Periaortic adipose tissue
PCAT	Pericoronary adipose tissue
PETVAS	PET vascular activity score
PET-CT	Positron emission tomography computerized tomography
PET-MRI	PET-magnetic resonance imaging
PGA	Physician global assessment
PLR	Platelet-to-lymphocyte ratio
PROMs	Patient-reported outcome measures
PTX-3	Pentraxin-related protein
RAG-1	Recombination activating gene 1
RCTs	Randomized controlled trials
RDW	Red cell distribution width
SAA	Serum amyloid A
SAP	Serum amyloid P
SNPs	Single nucleotide polymorphisms
SST2	Somatostatin receptor subtype 2
SUVmax	Maximum standardized uptake values
SUVmean	Mean standardized uptake values
TADS	Takayasu Arteritis Damage Score
TAK	Takayasu arteritis
TBR	Target-to-blood pool ratio
TNF	Tumor necrosis factor
TIMP-1	Tissue inhibitor of metalloproteinases 1
VCAM-1	Vascular cell adhesion molecule 1
VDI	Vasculitis Damage Index
VEGF	Vascular endothelial growth factor
